# Supplementary material for: Allosteric regulation of glycogen breakdown by the second messenger cyclic di-GMP
Source: Nat Commun. 2022 Oct 3;13:5834. doi: 10.1038/s41467-022-33537-w (PMC9530166; doi:10.1038/s41467-022-33537-w)
Supplement: Supplementary file 1 — Supplementary Information [file 41467_2022_33537_MOESM1_ESM.pdf]

Supplementary Information

Allosteric regulation of glycogen breakdown by the second messenger cyclic di-GMP

Maria A. Schumacher[1*§], Mirka E. Wörmann[2,3§], Max Henderson[1], Raul Salinas[1], Andreas Latoscha[2], Mahmoud M. Al-Bassam[4], Kumar Siddharth Singh,[5] Elaine Barclay[6], Katrin Gunka[5] and Natalia Tschowri[5*]

[1]*Department of Biochemistry, Duke University School of Medicine, Durham, NC 27710, USA*
[2]*Institute for Biology / Microbiology, Humboldt-Universität zu Berlin, 10115 Berlin, Germany*
[3]*Present address: Bundesinstitut für Risikobewertung, Diedersdorfer Weg 1, 12277 Berlin*
[4]*Department of Pediatrics, University of California, San Diego, La Jolla, California 92093, USA*
[5]*Institute of Microbiology, Leibniz Universität Hannover, 30419 Hannover, Germany*
[6]*Department of Cell and Developmental Biology, John Innes Centre, Norwich Research Park, Norwich NR4 7UH, UK*

§ These authors contributed equally to this work
*Correspondence: tschowri@ifmb.uni-hannover.de or maria.schumacher@duke.edu

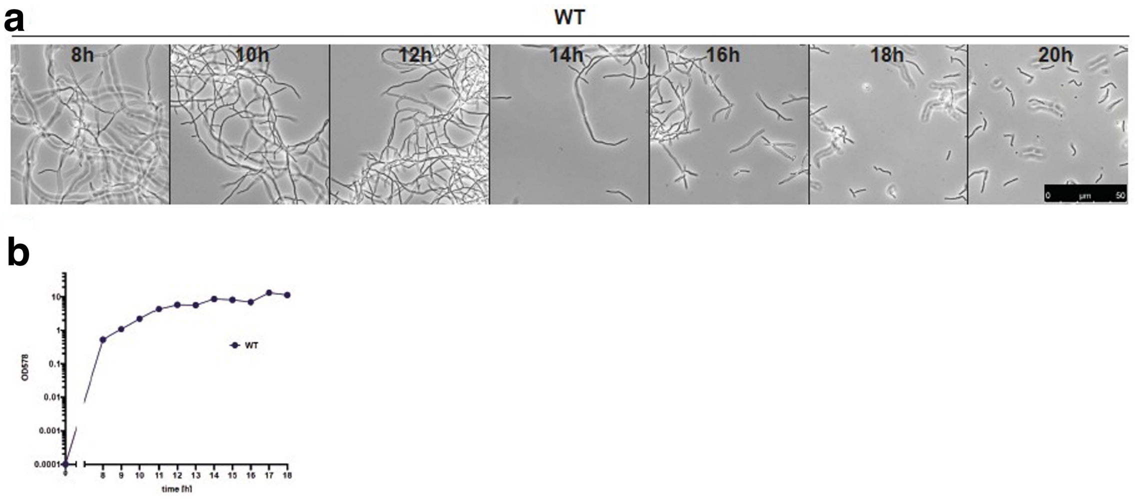

Supplementary Fig. 1: Morphology and growth of *S. venezuelae* during incubation in liquid Maltose-Yeast extract-Malt (MYM) extract medium. 100 ml MYM were inoculated with spores at a final concentration of 10^6 CFU/mL and incubated at 30°C and 170 rpm. **(a)** Cells were imaged using the Leica DM2000 LED microscope at 100× magnification. Initiation of fragmentation was observed after 12-14 h of incubation. After 16-18 h of growth, spores were detectable. **(b)** Optical density of *S. venezuelae* cultures was measured throughout the growth at 578 nm. Three biological replicates were analyzed and representative images are shown. Source data are provided in the Source data file.

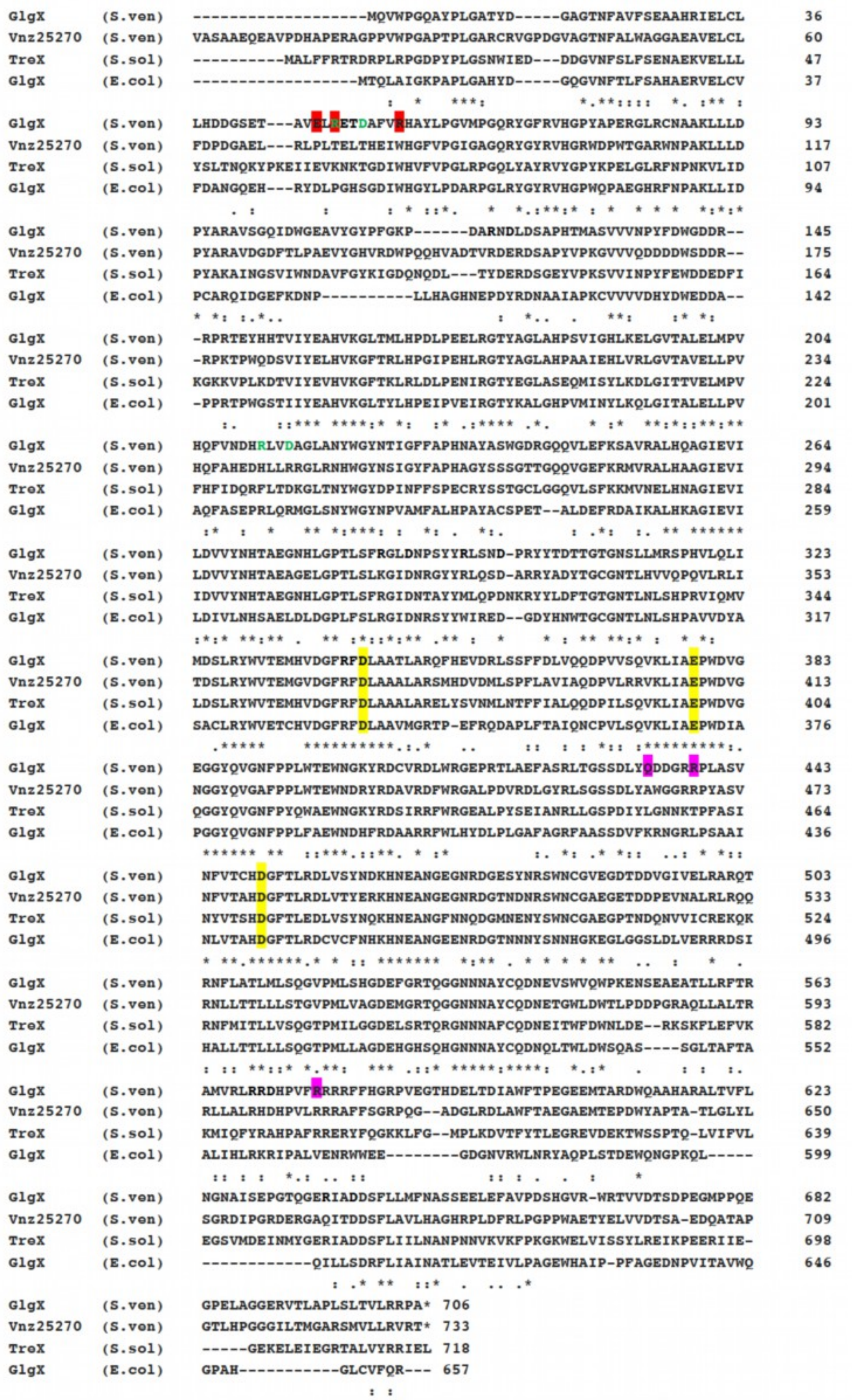

Supplementary Fig. 2: Protein alignment between GlgX (*S. venezuelae*), Vnz25270 (*S. venezuelae*), TreX (*S. solfataricus*) and GlgX (*E. coli*). The RxxD motifs swapped to DxxR in the *S. venezuelae* GlgX by mutagenesis are shown in green. Amino acids involved in c-di-GMP binding in the N-terminus of GlgX are highlighted in red, residues making contacts to c-di-GMP in the C-terminus of GlgX are marked in pink. Active site residues are shown in yellow. The alignment was generated using Clustal 2.1 and modified manually. An * (asterisk) indicates positions which are conserved. A colon (:) indicates conservation between residues of strongly similar properties. A period (.) indicates conservation of weakly similar properties.

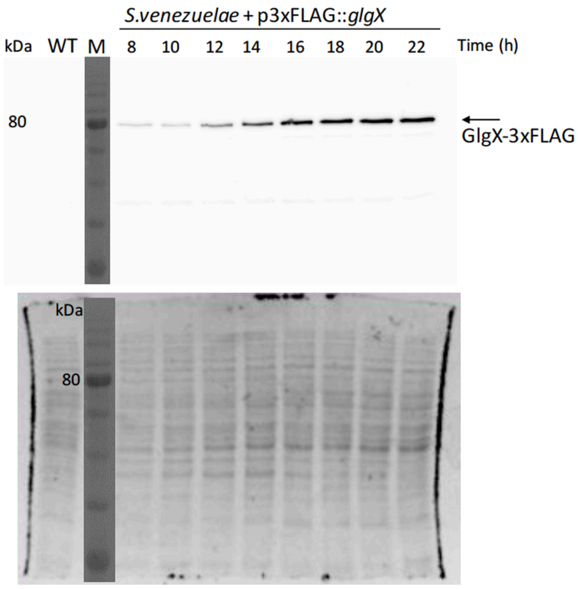

Supplementary Fig. 3: GlgX-FLAG expression during *S. venezuelae* developmental cycle. Protein samples were harvested after indicated time of growth and 20 µg total protein were used for Western blotting with the anti-FLAG antibody. *S. venezuelae* WT was used as negative control. The FLAG tagged GlgX is marked by an arrow. Sizes of proteins standards run in parallel are shown on the left of the panel. The lower parts of panel serves as a loading control showing proteins that were transferred onto the membrane and visualized using 2,2,2-Trichloroethanol and UV light. The experiment was performed twice and a representative image is shown.

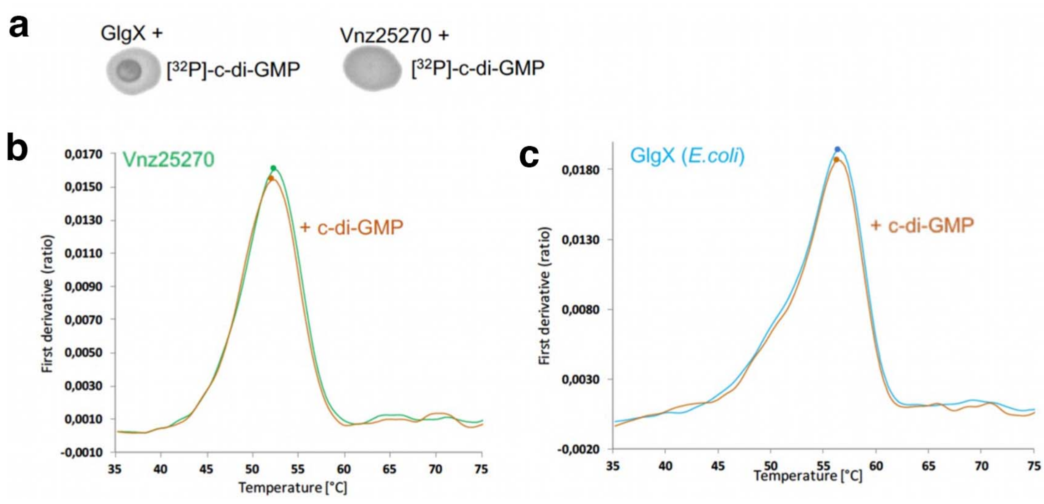

Supplementary Fig. 4: DRaCALA (a) and nanoDSF assays (b, c) showing that neither Vnz25270 from *S. venezuelae* nor GlgX from *E. coli* bind c-di-GMP. (**a**) 15 µg of purified *E. coli* His_6-GlgX or His_6-Vnz25270, respectively, were mixed with 0.5 µl of [^{32}P]-c-di-GMP (Hartmann Analytic GmbH, Braunschweig) in binding buffer (25 mM Tris pH 8.0, 150 mM NaCl, 2.5% (v/v) glycerol and 5 mM $MgCl_2$). 10 µL of the reactions were spotted on the same nitrocellulose membrane (Roth), air dried for 10 min and analyzed by phosphorimaging. (**b, c**) 10 µM purified His_6-Vnz25270 (**b**) or His_6-$GlgX_{E.coli}$ (**c**) in 25 mM Tris pH 8.0, 150 mM NaCl, 2.5% (v/v) glycerol and 5 mM $MgCl_2$ were incubated without or with (1 mM) c-di-GMP. Reactions were analyzed in a Tycho NT.6 (NanoTemper Technologies) device.

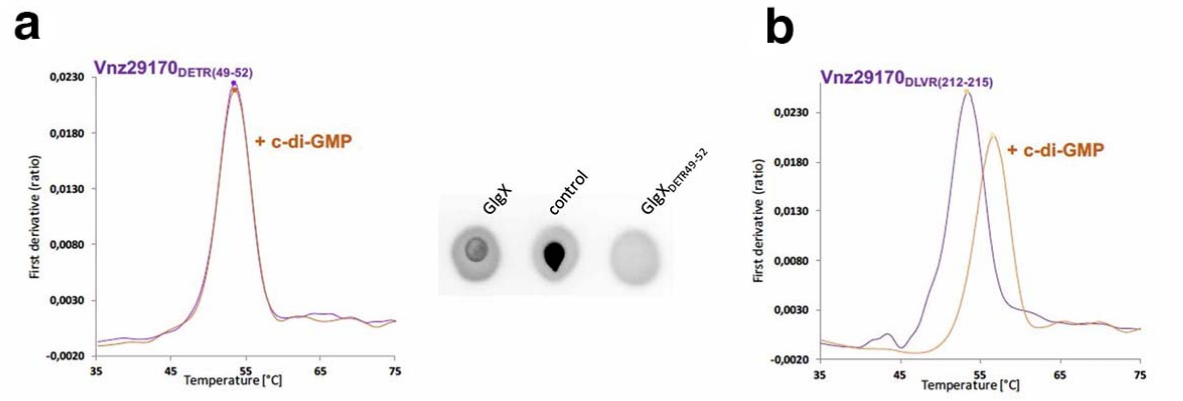

Supplementary Fig. 5: NanoDSF assays with mutagenized His$_6$-GlgX variants. (a) Swap of the D and R in the RETD motif (amino acids) 49-52 to DETR in GlgX abolishes c-di-GMP binding, left. This was confirmed by DRaCALA, right. For DRaCALA assays, 15 µg of purified *E. coli* His$_6$-GlgX or His$_6$-Vnz25270, respectively, were mixed with 0.5 µl of [^{32}P]-c-di-GMP (Hartmann Analytic GmbH, Braunschweig) in binding buffer (25 mM Tris pH 8.0, 150 mM NaCl, 2.5% (v/v) glycerol and 5 mM MgCl$_2$). 10 µl of the reactions were spotted on the same nitrocellulose membrane (Roth), air dried for 10 min and analyzed by phosphorimaging. **(b)** Changing the RLVD motif (amino acids 212-215) to DLVR still allows ligand binding.

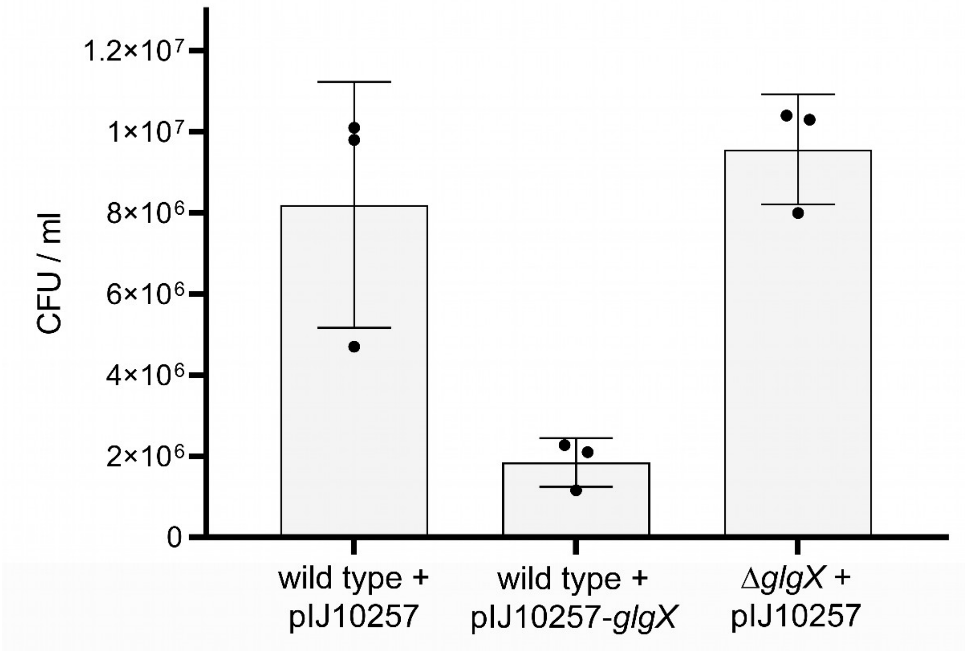

Supplementary Fig. 6: *S. venezuelae* overexpressing *glgX* from the integrative pIJ10257 vector forms less viable spores. Spores were collected from macrocolonies grown for 48 h at 30°C on on MYM-agar containing 0.03% maltose. Data are presented as the mean of biological replicates ± SD (n of replicates=12). Source data are provided as a Source data file.

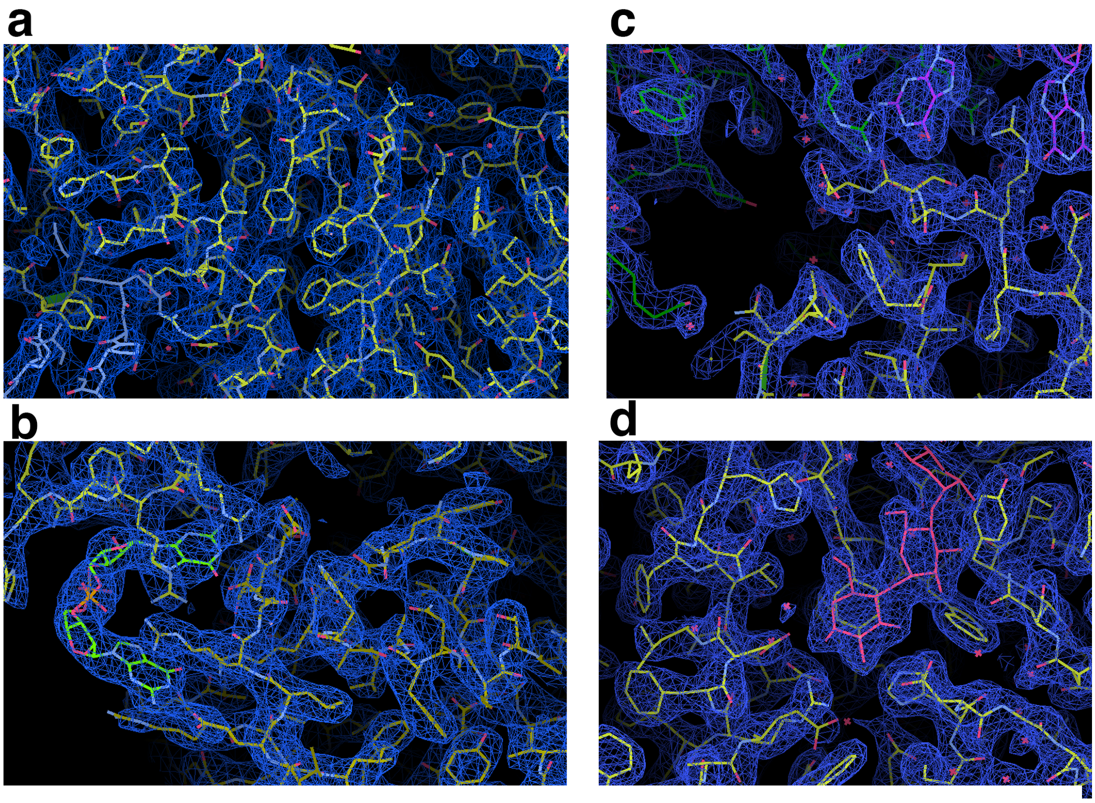

Supplementary Fig. 7: Representative sigma-A weighted 2mFo-DFc maps. Shown are sigma-A weighted 2mFo-DFc electron density maps for (**a**) apo GlgX. (**b**) GlgX-c-di-GMP and (**c, d**) GlgX-c-di-GMP-acarbose. The maps are contoured at 1 σ.

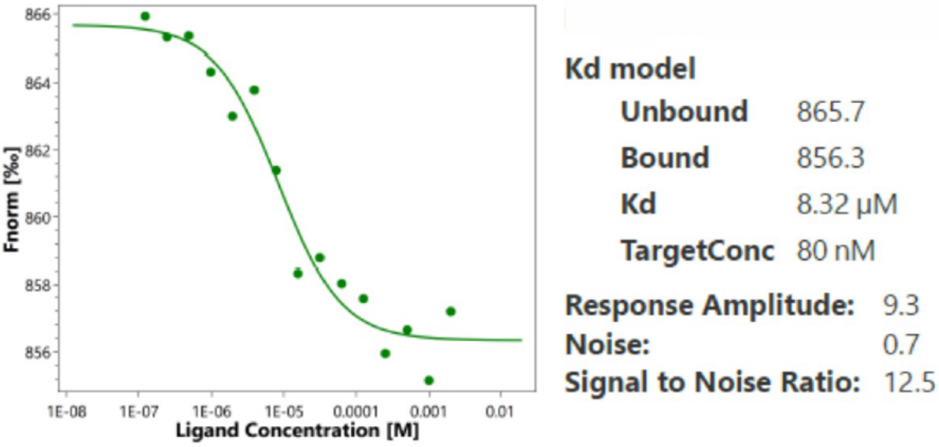

Supplementary Fig. 8: MST experiment examining the interaction between GlgX and c-di-GMP. Representative MST binding curve of c-di-GMP binding to WT GlgX. The resultant K_d was 8.3 µM. The MST experiment was done in replicate with n=2; We note the MST experiment is a supplementary analysis demonstrating c-di-GMP binding to GlgX; the GlgX-c-di-GMP interaction was also demonstrated by thermal shift, FP, DRaCALA and structural studies (multiple structures). Shown is a representative binding curve. Source data are provided in the Source data file.

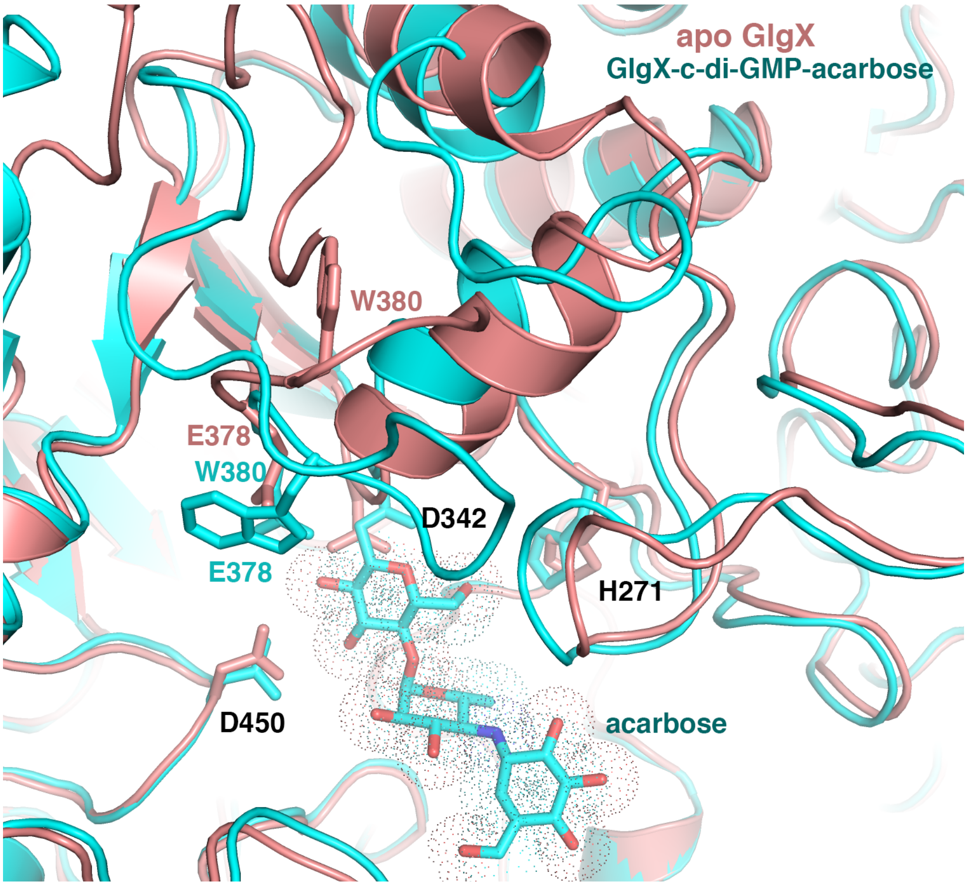

Supplementary Fig. 9: Close up of the active site comparing apo GlgX and GlgX-c-di-GMP-acarbose complex. The overlay underscores that key active site residues that comprise the site of hydrolysis (e.g. Trp380 and Glu378) are remodelled upon c-di-GMP binding, placing them in catalytically active positions.

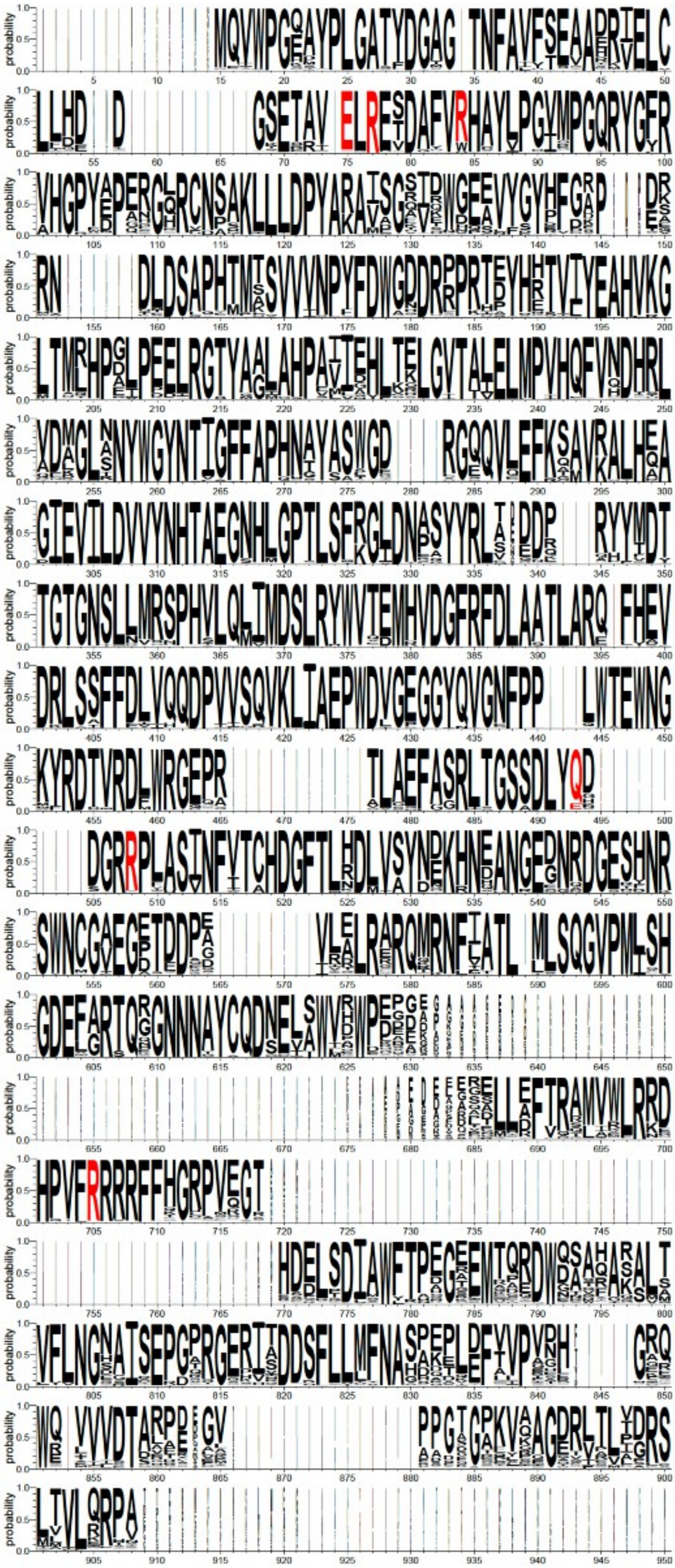

Supplementary Fig. 10: WebLogo showing c-di-GMP-binding residues in GlgX homologs. The WebLogo was generated using WebLogo 3 (http://weblogo.threeplusone.com/create.cgi) and is based on the alignment between GlgX homologs listed in Table S2. The width of the residues represents conservation, at positions with low conservations only thin lines are depicted. Residues involved in c-di-GMP-binding are highlighted in red.

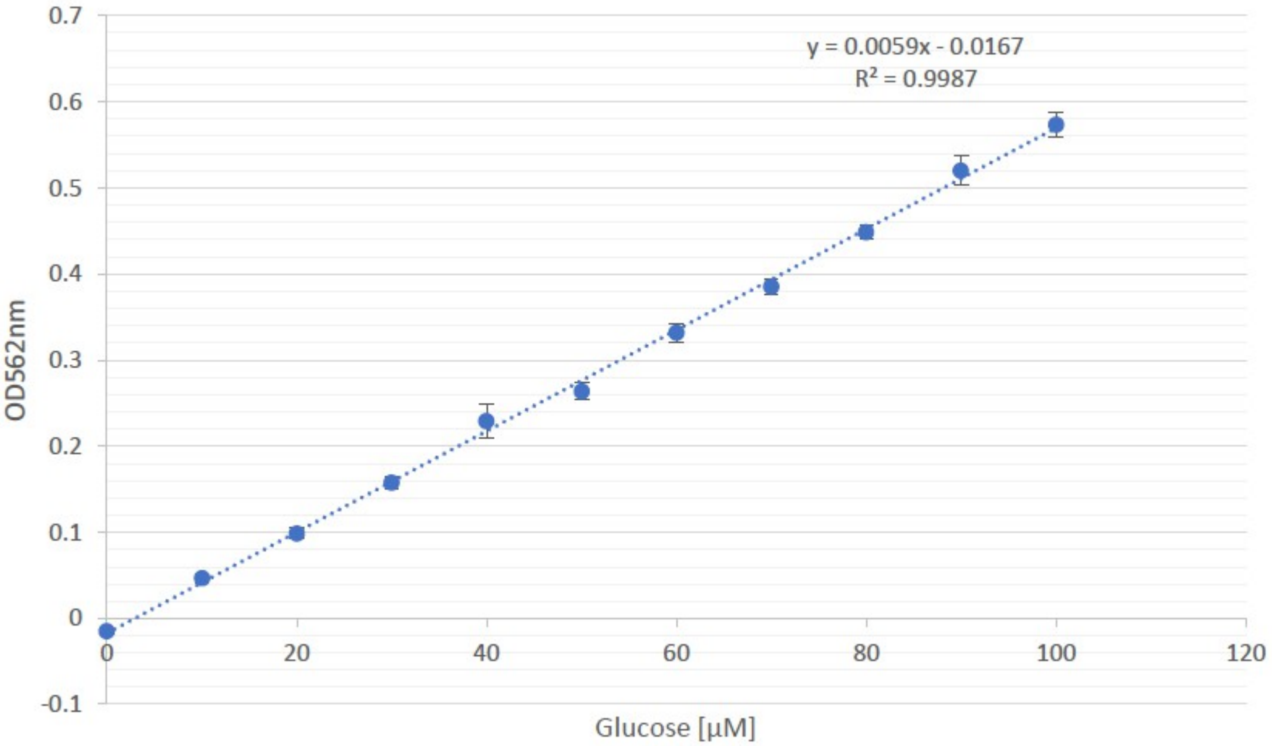

Supplementary Fig. 11: Standard curve for the determination of reducing ends of carbohydrates. Best-fit line obtained from 0 to 100 µM D-glucose. Data are presented as the mean of technical replicates ± SD. The experiment was performed three times, each time with two technical replicates. The data points represent the mean +/- SD of 6 data points. Source data are provided in the Source data file.

Supplementary Table 1: Data collection and refinement statistics: *Streptomyces venezuelae* GlgX structures

	GlgX-c-di-GMP	Apo GlgX	GlgX-c-di-GMP-Acarbose (pH 8.5)	GlgX-c-di-GMP-Acarbose (pH 4.5)
**Data collection**				
Pdb code	7U3A	7U39	7U3B	7U3D
Space group	C2	$P2_1$	$P2_1$	$P22_12_1$
Cell dimensions				
a, b, c (Å)	129.5,127.7,179.7	181.5,204.9,195.7	109.8,185.6,184.7	94.9,145.4,229.5
α, β, γ (°)	90.0,93.7,90.0	90.0,90.4,90.0	90.0,98.1,90.0	90.0,90.0,90.0
Resolution (Å)	89.67-3.34 (3.42-3.34)*	67.92 – 3.51 (3.55-3.51)	48.50-3.60 (3.78-3.60)	49.10-2.40 (2.53-2.40)
R_{sym} or R_{merge}	0.102 (0.323)	0.165 (0.438)	0.199 (0.780)	0.155 (1.045)
R_{pim}	0.095 (0.228)	0.116 (0.363)	0.170 (0.517)	0.070 (0.473)
$I / \sigma I$	6.2 (1.9)	5.5 (2.1)	5.5 (1.3)	11.0 (1.9)
Completeness (%)	99.5 (99.0)	83.5 (44.0)	98.2 (98.0)	99.9 (99.5)
Redundancy	2.0 (2.1)	1.7 (1.6)	1.7 (1.4)	6.5 (6.2)
CC(1/2)	0.977 (0.891)	0.969 (0.802)	0.923 (0.667)	0.995 (0.647)
**Refinement**				
Resolution (Å)	89.67-3.34	67.92-3.51	48.50-3.60	47.4-2.40
No. reflections	42293 (2854)	131795 (4072)	83323 (5752)	124127 (8764)
R_{work} / R_{free} (%)	20.5/27.5	21.1/27.6	23.6/30.3	17.2/23.4
R.m.s. deviations				
Bond lengths (Å)	0.003	0.004	0.011	0.004
Bond angles (°)	0.648	0.893	1.22	0.893
Ramachandran analyses				
Favored (%)	92.3	89.0	92.2	95.9
Disallowed (%)	0.00	0.00	0.00	0.00

*Values in parentheses are for highest-resolution shell.

Supplementary Table 2. Multiple sequence alignment of GlgX homologs: see xls spreadsheet.

Supplementary Table 3. Strains, plasmids and oligonucleotides used in this study

**Strains/ Collection number**	**Genotype or comments**	**Source or reference**
S. venezuelae		
NRRL B-65442	Wild type	(NCBI Reference Sequence: NZ_CP018074.1)
572	*S. venezuelae vnz29170 (glgX)::aac(3)IV*; Apr^R	This study
574	*S. venezuelae* $attB_{\Phi BT1}$::pIJ10257-*vnz29170 (glgX)*; Hyg^R	This study
721	*S. venezuelae vnz29170 (glgX)::aac(3)IV*; $attB_{\Phi BT1}$::pIJ10257-*vnz29170 (glgX)*; Apr^R, Hyg^R	This study
SVNT20	*S. venezuelae* $attB_{\Phi BT1}$::pIJ10257; Hyg^R	This study
576	*S. venezuelae* $attB_{\Phi BT1}$:: p3xFLAG_*vnz29170(glgX)*; Hyg^R	This study
722	*S. venezuelae vnz29170 (glgX)::aac(3)IV*; $attB_{\Phi BT1}$:: p3xFLAG -*vnz29170 (glgX)*; Apr^R, Hyg^R	This study
E. coli		
ET12567/pUZ8002	*dam*, *dcm*, *hsd*; Kan^R, Cm^R	1
BW25113/pIJ790	(Δ(*araD-araB*)*567*, Δ*lacZ4787*(::*rrnB-4*), *lacIp-4000*($lacI^Q$), λ-, *rpoS369*(Am), *rph-1*, Δ(*rhaD-rhaB*)568, *hsdR514*; Cm^R	2
C41(DE3)		Novagen
Rosetta 2 (DE3)	F^- *ompT hsdS*$_B$(r_B^- m_B^-) *gal dcm* (DE3) pRARE2 (Cam^R)	Novagen
**Plasmids/ Collection number**		
pIJ773	Plasmid template for amplification of the *apr-oriT* cassette for 'Redirect' PCR-targeting; Apr^R	3
pIJ790	Modified λRED recombination plasmid [*oriR101*] [*repA101*(ts)] *araBp-gam-be-exo*; Cm^R	3

pIJ10257 / 88	Plasmid integrating at the ϕBT1 *attB* attachment site containing the constitutive *ermE** promoter, HygR	4
pUZ8002	RP4 derivative with defective oriT; Kan^R	1
p3xFLAG	pIJ10770 derivative containing *3xFLAG* sequence downstream of MCS; Hyg^R	5
pET15b / 113	T7 expression vector; Amp^R	Novagen
pET15b_*glgX* (*E. coli*) / 294	Overexpression of His_6-*glgX* from *E. coli*, AmpR	This study
pET15b_*vnz29170 (glgX)* / 152	Overexpression of His_6-*vnz29170* (*glgX*), AmpR	This study
pET15b_*vnz25270* / 288	Overexpression of His_6- *vnz25270*, AmpR	This study
pET15b_*vnz29170 (glgX)*$_{D342A}$ / 355	Overexpression of His_6-*vnz29170* (*glgX*)$_{D342A}$, AmpR	This study
pET15b_*vnz29170 (glgX)*$_{E378A}$ / 356	Overexpression of His_6-*vnz29170* (*glgX*)$_{E378A}$, AmpR	This study
pET15b_*vnz29170 (glgX)*$_{D342A+E378A}$ / 425	Overexpression of His_6-*vnz29170* (*glgX*)$_{D342A+E378A}$, AmpR	This study
pET15b_*vnz29170 (glgX)*$_{DETR\ 49-52}$ / 418	Overexpression of His_6-*vnz29170* (*glgX*) $_{DETR\ 49-52}$, AmpR	This study
pET15b_*vnz29170 (glgX)*$_{DLVR\ 212-215}$ / 420	Overexpression of His_6-*vnz29170* (*glgX*) $_{DLVR\ 212-215}$, AmpR	This study
pIJ10257_*vnz29170 (glgX)* / 295	Expression of *vnz29170* (*glgX*) from the ϕBT1 *attB* attachment site controlled by the constitutive *ermE** promoter, HygR	This study
p3xFLAG_*vnz29170 (glgX)* / 256	Expression of *vnz29170* (*glgX*)-FLAG from the ϕBT1 *attB* attachment site controlled by the native promoter, HygR	This study
**Oligonucleotides (**Relevant restriction sites are underlined)		

**Name / MW Nr.**	**Sequence 5´-3´**
F_vnz27190_knockout / MW163	CTACACAACCCGGACAGAAGAGGAAGAGTCGTATCCATG ATTCCGGGGATCCGTCGACC
R_vnz27190_knockout / MW164	CGGTCCGGGTGCCGGCGCCCGTTCCCGTACGGAAGGTCA TGTAGGCTGGAGCTGCTTC
F_seq_vnz27190delete / MW165	ATTTCGTACACACTTCCCCCTC
R_seq_vnz27190delete / MW166	GAGTCCATGGCCTTCCTGAC
F_SVEN_5898_NdeI / MW87	ttccatatgCAGGTCTGGCCGGGTCAGGCG
R_vnz29170_HindIII / MW187	ttcaagcttCGTTCCCGTACGGAAGGTCAGGCC
F_vnz29170_FlagTag_HindIII / MW171	ttcaagcttGTCCGTCCCCTCGTTCCCTTCGCTCCCAGCGGCC
R_vnz29170_FlagTag_XhoI / MW172	gttcctcgagGGCCGGCCGCCGCAGAACGGTCAGGC
R_SVEN_5898_BamHI / MW88	ttcggatccCGTTCCCGTACGGAAGGTCAGGCC
F_NdeI_vnz25270 / MW185	ttccatatgGCGAGCGCAGCCGAGCAGGAGGCGGTAC
R_BamHI_vnz25270 / MW186	ttcggatccGTCCGGGCGTTCAGGTCCTGACCCGGAGCAG
F_vnz29170_D342A / MW197	TTCCGCTTCGCCCTGGCGGCCACCCTGGCCCGCCAG
R_vnz29170_D342A / MW198	GGTGGCCGCCAGGGCGAAGCGGAAACCGTCCACGTGCATCTCGGTC
F_vnz29170_E378A / MW199	CTGATCGCCGCCCCCTGGGACGTCGGCGAGGGCGGC
R_vnz29170_E378A / MW200	GACGTCCCAGGGGGCGGCGATCAGTTTCACCTGGCTGACCACCGG

F_vnz29170_front_GA / MW213	GGCAGCCATATGCAGGTCTGGCCGG
R_vnz29170_DETR_49-52aa / MW205	GAACGCGCGGGTCTCGTCTAGTTCCACCGCCGTCTCCGAGCCG
F_vnz29170_DETR_49-52aa / MW206	GAACTAGACGAGACCCGCGCGTTCGTGCGGCACGCCTATCTGC
R_vnz29170_back_GA / MW214	GCAGCCGGATCCCGTTCCCGTACG
R_vnz29170_DLVR_212-215aa / MW209	GCCCGCGCGCACCAGGTCGTGGTCGTTGACGAACTGGTGGACGGGCATC
F_vnz29170_DLVR_212-215aa / MW210	GACCACGACCTGGTGCGCGCGGGCCTCGCCAACTACTGGGGC
F_pET15b_vnz29170_GA / MW215	CGTACGGGAACGGGATCCGGCTGCTAAC
R_pET15b_vnz29170_GA / MW216	CGGCCAGACCTGCATATGGCTGCCG
F_NdeI_glgX_Ecoli / MW190	ttccatatgACACAACTCGCCATTGGCAAACC
R_BamHI_glgX_Ecoli / MW191	ttcggatccCGCGCCAACATTAAGTGATCGTTCTTCTC

Supplementary References

1. Paget, M.S., Chamberlin, L., Atrih, A., Foster, S.J. & Buttner, M.J. Evidence that the extracytoplasmic function sigma factor sigmaE is required for normal cell wall structure in *Streptomyces coelicolor* A3(2). *J. Bacteriol.* **181**, 204-211 (1999).

2. Datsenko, K.A. & Wanner, B.L. One-step inactivation of chromosomal genes in *Escherichia coli* K-12 using PCR products. *Proc. Natl. Acad. Sci. USA* **97**, 6640-6645 (2000).

3. Gust, B., Challis, G.L., Fowler, K., Kieser, T. & Chater, K.F. PCR-targeted *Streptomyces* gene replacement identifies a protein domain needed for biosynthesis of the sesquiterpene soil odor geosmin. *Proc. Natl. Acad. Sci. USA* **100**, 1541-1546 (2003).

4. Hong, H.J., Hutchings, M.I., Hill, L.M. & Buttner, M.J. The role of the novel Fem protein VanK in vancomycin resistance in *Streptomyces coelicolor*. *J. Biol. Chem.* **280,** 13055-13061 (2005).

5. Al-Bassam, M.M., Haist, J., Neumann, S.A., Lindenberg, S. & Tschowri, N. Expression Patterns, Genomic Conservation and Input Into Developmental Regulation of the GGDEF/EAL/HD-GYP Domain Proteins in *Streptomyces*. *Front. Microbiol.* **9**, 2524 (2018).
