# Supplementary material for: Allosteric regulation of glycogen breakdown by the second messenger cyclic di-GMP
Source: Nat Commun. 2022 Oct 3;13:5834. doi: 10.1038/s41467-022-33537-w (PMC9530166; doi:10.1038/s41467-022-33537-w)
Supplement: Supplementary file 3 — Description of Additional Supplementary Files [file 41467_2022_33537_MOESM3_ESM.pdf]

Description of Additional Supplementary Files

Supplementary Movie 1: Ribbon diagram of the S. venezuelae GlgX-c-di-GMP-acarbose complex. One GlgX subunit is colored cyan and the other salmon. The c-di-GMP (whilte) and acarbose molecules (yellow) are shown as sticks. Close up animations are provided for the c-di-GMP binding site with hydrogen bond interactions indicated as yellow dashed lines between c-di-GMP and GlgX side chain atoms.
