# Supplementary material for: Allosteric regulation of glycogen breakdown by the second messenger cyclic di-GMP
Source: Nat Commun. 2022 Oct 3;13:5834. doi: 10.1038/s41467-022-33537-w (PMC9530166; doi:10.1038/s41467-022-33537-w)
Supplement: Supplementary file 6 — Reporting Summary [file 41467_2022_33537_MOESM6_ESM.pdf]

nature portfolio

Corresponding author(s): Maria A. Schumacher/Natalia Tschowri

Last updated by author(s): September 9, 2022

Reporting Summary

Nature Portfolio wishes to improve the reproducibility of the work that we publish. This form provides structure for consistency and transparency in reporting. For further information on Nature Portfolio policies, see our Editorial Policies and the Editorial Policy Checklist.

Statistics

For all statistical analyses, confirm that the following items are present in the figure legend, table legend, main text, or Methods section.

n/a	Confirmed	
[ ]	[x]	The exact sample size (*n*) for each experimental group/condition, given as a discrete number and unit of measurement
[ ]	[x]	A statement on whether measurements were taken from distinct samples or whether the same sample was measured repeatedly
[x]	[ ]	The statistical test(s) used AND whether they are one- or two-sided<br>*Only common tests should be described solely by name; describe more complex techniques in the Methods section.*
[x]	[ ]	A description of all covariates tested
[x]	[ ]	A description of any assumptions or corrections, such as tests of normality and adjustment for multiple comparisons
[ ]	[x]	A full description of the statistical parameters including central tendency (e.g. means) or other basic estimates (e.g. regression coefficient) AND variation (e.g. standard deviation) or associated estimates of uncertainty (e.g. confidence intervals)
[x]	[ ]	For null hypothesis testing, the test statistic (e.g. *F*, *t*, *r*) with confidence intervals, effect sizes, degrees of freedom and *P* value noted<br>*Give P values as exact values whenever suitable.*
[x]	[ ]	For Bayesian analysis, information on the choice of priors and Markov chain Monte Carlo settings
[x]	[ ]	For hierarchical and complex designs, identification of the appropriate level for tests and full reporting of outcomes
[x]	[ ]	Estimates of effect sizes (e.g. Cohen's *d*, Pearson's *r*), indicating how they were calculated

Our web collection on statistics for biologists contains articles on many of the points above.

Software and code

Policy information about availability of computer code

Data collection	XDS 0.6, DigitalMicrograph (version 3.5) from Gatan
Data analysis	Phenix 1.19, Coot 0.9.6, PyMOL (Version 2.0), MolProbity 4.5.1, KaleidaGraph 4.5, serial # 8011073 (Synergy Software), BLAST 2.12.0, PATRIC (version 3.6.12)

For manuscripts utilizing custom algorithms or software that are central to the research but not yet described in published literature, software must be made available to editors and reviewers. We strongly encourage code deposition in a community repository (e.g. GitHub). See the Nature Portfolio guidelines for submitting code & software for further information.

Data

Policy information about availability of data

All manuscripts must include a data availability statement. This statement should provide the following information, where applicable:

- Accession codes, unique identifiers, or web links for publicly available datasets
- A description of any restrictions on data availability
- For clinical datasets or third party data, please ensure that the statement adheres to our policy

Coordinates and structure factor amplitudes have been deposited with the Protein Data Bank under the accession codes 7U3A, 7Us9, 7U3B, and 7U3D.

Field-specific reporting

Please select the one below that is the best fit for your research. If you are not sure, read the appropriate sections before making your selection.

[x] Life sciences [ ] Behavioural & social sciences [ ] Ecological, evolutionary & environmental sciences

For a reference copy of the document with all sections, see nature.com/documents/nr-reporting-summary-flat.pdf

Life sciences study design

All studies must disclose on these points even when the disclosure is negative.

Sample size	X-ray and BCA datasets were collected on independently prepared samples. For X-ray data sample size was determined by reaching high completeness. For c-di-GMP extraction and glycogen quantification 3 independent samples were used. For FP and enzyme assays, no sample size calculations were performed. Sample size for FP was based on the range of expected Kd (and the required volumes that were dictated by the equipment). Sample size for enzyme assays were similarly based on standard volumes of assays and range of measured parameters. These sizes were adequate based on the consistency of measurable differences between groups. For FP, all experiments were done in triplicate and for enzyme assays, measurements were done in at least triplicate. These sample sizes allowed for robust statistical analyses.
Data exclusions	No data were excluded from experiments and the crystallographic data were processed with XDS using the default parameters.
Replication	Crystallographic data were collected on one crystal per data set. TTriplicates were performed for all growth experiments and phenotypical analysis, FP, BCA, c-di-GMP and glycogen quantification assay studies. For other data, at least two replicates were performed. All attempts at replication were successful. X-ray structural coordinates were tested and validated by the MolProbity server before deposition to the Protein Data Bank and independent validation reports were then obtained from the Protein Data Bank upon data deposition. For cell based assays each experiment was performed in at least two biological replicates. All replication attempts were successful. For each replicate, experimental and control groups were imaged or measured, processed and analyzed together.
Randomization	Crystallography samples were independent of each other. Data were collected for each crystal form at least twice with the electron density maps confirming the presence of ligands (for ligand bound) or absence of ligands and altered conformations (for apo structure). For FP, DRaCALA, thermal shift assays and enzyme assays all data were included in at least triplicate to ensure replication. Randomization was not applicable for the biochemical assays as standard procedures were applied. For cell based assays strains for different experiments were selected randomly for inoculation from plate. All strains were grown under similar conditions and were therefore equivalent at the start of the experiment. The observed differences are due to the genotype of the analyzed strains.
Blinding	Researchers were not blinded during crystallographic or other data collection or analysis. Prior information regarding the samples is necessary to guide data collection and processing. However, data were collected for each crystal form at least twice with the electron density maps confirming the presence of ligands (for ligand bound) or absence of ligands and altered conformations (for apo structure).For cell based assays, strains were grown under similar conditions and were therefore equivalent at the start of the experiment.

Reporting for specific materials, systems and methods

We require information from authors about some types of materials, experimental systems and methods used in many studies. Here, indicate whether each material, system or method listed is relevant to your study. If you are not sure if a list item applies to your research, read the appropriate section before selecting a response.

Materials & experimental systems

n/a	Involved in the study	
[ ]	[x]	Antibodies
[x]	[ ]	Eukaryotic cell lines
[x]	[ ]	Palaeontology and archaeology
[x]	[ ]	Animals and other organisms
[x]	[ ]	Human research participants
[x]	[ ]	Clinical data
[x]	[ ]	Dual use research of concern

Methods

n/a	Involved in the study	
[x]	[ ]	ChIP-seq
[x]	[ ]	Flow cytometry
[x]	[ ]	MRI-based neuroimaging

Antibodies

Antibodies used	monoclonal anti-FLAG M2 antibody: Sigma Aldrich F3165; HRP-conjugated anti-mouse antibody: GE Healthcare NA931.
Validation	Validation of the commercially available anti-FLAG M2 antibody was based on technical data sheet provided by Sigma Aldrich: anti-FLAG M2: https://www.sigmaaldrich.com/certificates/COFA/F3/F3165/F3165-BULK________SLBW9109__.pdf As a negative control, crude extract from S. venezuelae wild type was used.
